# Nutrient intake and adherence to the Nordic nutrition recommendations in a Swedish cohort with abdominal obesity

**DOI:** 10.1177/02601060221105751

**Published:** 2022-06-03

**Authors:** Åsa Sedin, Mona Landin-Olsson, Lieselotte Cloetens

**Affiliations:** 1 Biomedical nutrition, Pure and Applied Biochemistry, 5193 Lund University, Lund, Sweden; 2 Department of Clinical Sciences, 5193 Lund University, Lund, Sweden; 3 Department of Endocrinology, 59564 Skåne University Hospital, Lund, Sweden

**Keywords:** Abdominal obesity, Nordic nutrition recommendations, adherence, dietary fat quality, public health

## Abstract

**Background:**

The Nordic Nutrition Recommendations (NNR) are developed to promote public health and to prevent food-related diseases such as obesity and cardiovascular diseases.

**Objective:**

To investigate the nutrient intake and adherence to the NNR in a Swedish cohort with abdominal obesity.

**Design:**

Dietary intake data were collected using 3-day food diaries and anthropometry and clinical chemistry parameters were measured at baseline of a long-term intervention studying weight-loss management.

**Results:**

Eighty-seven subjects with abdominal obesity successfully completed a 3-day food diary. Twelve of these subjects were excluded for further analysis due to implausible low-energy reporting. The remaining 75 subjects (76% females) had mean age of 52.3 ± 10.1 years and a mean body mass index of 34.3 ± 3.1 kg/m^2^. Mean total fat intake (41.2 ± 7.0E%) was exceeded by 56% of the sample size compared to the maximum recommended intake (RI) of 40E%, whereas mean carbohydrate intake (40.4 ± 8.0E%) was lower than the RI (45–60E%). The intake of saturated fatty acids was high compared to the NNR with only 2 women and none of men reported intakes within the RI of <10 E%. Adherence to the RI for dietary fibre was very low (16.0% and 13.3% when expressed as g/d and g/MJ, respectively). Analyses of micronutrient intake showed lowest adherences for vitamin D and sodium.

**Conclusions:**

The nutrient intake in our subjects compared to NNR was rather low with a high total fat intake, particularly too high intake of saturated fatty acids, high salt consumption, and very low dietary fibre and vitamin D intake. More effort is clearly needed to promote healthy dietary habits among subjects with obesity.

## Introduction

The Nordic Ministry Council publishes recommendations for nutrient intake in the Nordic countries, devised in the Nordic nutrition recommendations (NNR) (Nordic Nutrition Recommendations, 2012). NNR are primarily valid for healthy individuals and are used for e.g., developing nutrition policies, nutritional educational programs, and food regulations. One of the main aims of NNR is to advise a diet that is nutritionally adequate and minimises lifestyle related diseases, including obesity, cardiovascular disease (CVD), type 2 diabetes, cancer, and osteoporosis.

In 2010–2011, a large Swedish national governmental survey was performed in 2268 adults (18–80y) to study the dietary intake and food habits of the Swedish population (Amcoff et al., 2012). It was concluded that the adherence to the recommended dietary intake is low. It was reported that the recommended intake of fruits and vegetables is followed by only 20%, the intake of fish by 30% and the intake of the dietary fibre by 30%. Forty percent of the subjects consume too much sugar, and 80% had too high intake of saturated fatty acids (SFA). The same report noted that women in general have healthier dietary habits than men.

The acceptance of a healthy diet by the population is of importance for its implementation. In the Nordic countries, the Nordic diet is recommended to the Nordic population. The Nordic diet is mainly based on the consumption of local foods like oats, barley, root vegetables, berries, fish, and rapeseed oil (Mithril et al., 2012). This diet is rich in dietary fibre, unsaturated fats and foods with low glycemic index, and a low intake of saturated fats. The Nordic diet has been shown to have health-promoting effects including improved blood lipid profiles, lowered blood pressure and low-grade inflammation markers (Adamsson et al., 2011; Lankinen et al., 2016; Poulsen et al., 2014; Uusitupa et al., 2013). Danish research has shown that people are interested to consume the Nordic diet due its high eating qualities but that cultural and social barriers must be overcome to reach a higher degree of acceptance (Micheelsen et al., 2013; Micheelsen et al., 2014). Besides its health effects, a Nordic diet is also attractive as an environmentally healthy diet since it is based upon the use of local foods and food products (Meltzer et al., 2019).

Since there is a strong correlation between the dietary quality, obesity and chronic diseases (Organisation for Economic Co-operation and Development, 2018), increasing the knowledge about the dietary habits of subjects with obesity is of importance in order to re-adjust existing governmental health programs concerning dietary intervention. However, the information is very limited on how subjects with obesity adhere to the nutrition recommendations in the Nordic countries. In a study with a Nordic population with the metabolic syndrome (Mets), it was observed that more than 80% of the subjects consumed more SFA than the recommended intake level (RI), 70% had too low intake of polyunsaturated fatty acids (PUFA), around 80% had reduced intake of dietary fibre, almost 20% showed an intake of vitamin D that was below the lower level of intake, and the overall consumption of sodium was too high according to the RI (Jonsdottir et al., 2013).

In the present study, we evaluated the nutrient intake of adults with abdominal obesity living in Southern Sweden and to which extent it adheres to NNR.

## Materials and methods

The present study is part of a project investigating long-term weight maintenance after weight loss. The data presented in here represents the baseline data from that randomised controlled parallel intervention study. The number of study subjects recruited to the study was determined by power calculations using an α-level of 0.05 and a β-level of 0.80 for body weight and sagittal abdominal diameter (SAD) as primary endpoint.

### Ethical issues

This study was conducted according to the guidelines laid down in the Declaration of Helsinki and all procedures involving research study participants were approved by The Regional Ethical Review Board in Lund, Sweden (Dnr 2016/377). Written informed consent was obtained from all subjects.

### Subjects

Subjects with abdominal obesity were recruited by advertising in local media between August 2016 and October 2017. The main inclusion criteria were: age 25–67 years, body mass index (BMI) 30–40 kg/m^2^, SAD of >22 cm for men and >20 cm for women and waist circumference >102 cm for men and >88 cm for women. The main exclusion criteria included chronic diseases, alcohol abuse, diagnosed diabetes, adherence to extreme diets (e.g. Atkins, low-carb/high-fat diet, or vegetarian diets) and an estimated change of body weight of >3% three months prior to the screening visit. Before enrolment, a screening examination was carried out in which a routine health examination and a collection of background dietary intake took place.

### Study design and analyses

The study subjects were instructed to fill in a food diary following their regular eating habits and pattern. All intake of food and drink was registered for three consecutive days, including one weekend day. Food intake was reported either as weight or portion size. The mean nutrient content of the diet was calculated using the software DietistNETPro (version 18.11.18), based on the Swedish Food Database and data from food manufacturers. Subjects with low-energy reporting according to the Goldberg cut-off were excluded (Black, 2000). The Goldberg cut-off value calculated for our cohort was 0.91. This means that low-energy reporters were defined as a mean energy intake to basal metabolic rate ratio < 0.91.

The extent of adherence to NNR was calculated as the percentage of subjects that was within the range of the recommended intake (RI) for macronutrients and the percentage of subjects that reached the average requirements (AR) levels for micronutrients. RI is the amount of a nutrient needed to maintain a good nutritional status in healthy individuals (Nordic Nutrition Recommendations, 2012). AR is defined as the lowest intake level of a nutrient over a long-term that will maintain a defined level of nutritional status in a subject (Nordic Nutrition Recommendations, 2012).

Nutrient density of micronutrients was also calculated as the amount of micronutrient per energy content (per 10 MJ). Nutrient density is used for the planning of diets for a group of subjects within a heterogenous age and sex distribution (Nordic Nutrition Recommendations, 2012).

After an overnight fasting, the study subjects visited the study centre for blood sampling, blood pressure measurement and measurement of anthropometric parameters. Clinical chemistry parameters (total cholesterol, total triglycerides, high-density lipoprotein (HDL) cholesterol, low-density lipoprotein (LDL) cholesterol and glucose) were measured at Skåne University Hospital (Lund, Sweden) according to standard analytical procedures. Blood pressure and anthropometric measurements were measured by standardised methods and by experienced personnel. Blood pressure was measured using automatic devices in sitting position and in duplicate. Body weight was measured using an electronic scale with the subjects wearing light clothing and without shoes. Height was measured without shoes, and BMI was calculated as weight (kg) divided by the square of height (m). Waist circumference was measured at the level midway between the lateral lower rib margin and the iliac crest SAD was measured using an abdominal calliper (Holtain Ltd, UK) after a normal expiration while in the supine position with bend knees. Bio-electrical bioimpedance analysis (TANITA BC-418 MA(III)) was performed to measure body composition. After entering gender, age, height and physical activity level, the instrument´s software was used to obtain values of body fat (kg and %), fat-free mass (kg), total body water (kg) and basal metabolic rate (kcal).

The International Diabetes Federation (IDF) criteria were used to calculate the number of subjects having the MetS (Alberti et al., 2013). Subjects having the MetS were defined as having abdominal obesity (waist circumference ≥102 cm for men and ≥ 88 cm for women) and at least two of the following criteria: elevated triglycerides (≥ 1.7 mmol/L), reduced HDL-cholesterol (< 1.0 mmol/L in males and < 1.3 mmol/L in females), elevated blood pressure (systolic ≥ 130 mmHg and/or diastolic ≥ 85 mmHg) and elevated fasting glucose (≥ 5.6 mmol/L). Drug treatment for these MetS characteristics were used as an alternate indicator of the respective risk factors.

### Statistics

Statistical analysis was performed with MINITAB Statistical Software (version 17.1.0, Minitab Inc). Normality testing was performed with the Anderson-Darling normality test. Results are expressed as mean and standard deviation. T-tests were used to compare anthropometric and clinical chemistry data and nutrient intake between genders when the variables were normally distributed. Mann-Whitney tests were applied when the variables were non-normally distributed. A p-value of <0.05 was set as statistically significant.

## Results

A total of 102 subjects were recruited to a screening visit. Eighty-seven subjects fulfilled the criteria and filled in the 3-day food diary. Twelve subjects (15%), whereof nine men, were excluded from further data analysis due to improbable low-energy reporting as calculated by the Goldberg cut-off. The BMI between low-energy reporters (35.5 ± 2.7 kg/m^2^) and adequate energy reporters (34.3 ± 3.1 kg/m^2^) was not significantly different. Of the remaining subjects, four (5%) were treated for high cholesterol levels, and 15 (20%) subjects were treated for high blood pressure.

Anthropometric and clinical chemistry characteristics are shown in Table 1. Overall, 45.6% of the women and 38.9% of the men had the MetS according to the IDF criteria.

**Table 1.** Characteristics of study subjects (n = 75). Data are expressed as mean and SD.

	All (n = 75)		Women (n = 57)		Men (n = 18)		
	mean	SD	mean	SD	mean	SD	p-value
Age (years)	52.3	10.1	52.0	10.4	52.9	9.4	0.852
Body weight (kg)	98.7	13.5	93.6	9.7	114.6	11.6	<0.001
BMI (kg/m^2^)	34.3	3.1	34.2	3.0	34.8	3.4	0.468
Waist circumference (cm)	107.8	10.1	104.3	8.4	118.8	6.3	<0.001
Sagital abdominal diameter (cm)	26.0	2.5	25.2	2.1	28.6	1.9	<0.001
Body fat (%)	42.0	5.8	44.7	3.3	33.3	2.7	<0.001
Systolic blood pressure (mm Hg)	121.5	14.4	120.4	14.7	125.0	13.0	0.216
Diastolic blood pressure (mm Hg)	76.8	9.0	75.6	9.0	80.6	8.4	0.041
Total cholesterol (mmol/l)	5.4	0.9	5.5	0.9	5.4	0.9	0.881
Total triglycerides (mmol/l)	1.4	0.8	1.4	0.8	1.6	1.0	0.199
HDL-cholesterol (mmol/l)	1.5	0.5	1.5	0.5	1.2	0.3	<0.001
LDL-cholesterol (mmol/l)	3.7	0.8	3.7	0.8	3.8	0.8	0.491
Glucose (mmol/l)	5.6	0.6	5.6	0.6	5.7	0.7	0.577
Smoking (%)	13.3		15.8		0.05		0.519
Metabolic syndrome (%)	44.0		45.6		38.9		0.673

p-value denotes the difference between women and men. A p-value <0.05 is set as statistically significant.

### Macronutrients

Table 2 shows mean daily energy and macronutrient intake and the adherence to the recommended daily intake according to NNR. The mean energy intake of our subjects was 2329 ± 547 kcal, which is about 18% higher compared to the results from the Swedish national governmental survey (i.e. 1978 ± 623 kcal/d) (Amcoff et al., 2012).

**Table 2.** Mean daily energy and macronutrient intake in relation to NNR values.

Energy and macronutrients	All (n = 75)				Women (n = 57)				Men (n = 18)		
	NNR (RI)	mean	SD	within RI (%)	mean	SD	within RI (%)	mean	SD	within RI (%)	p-value
Total energy (kcal)		2329	547	-	2215	508	-	2692	520	-	0.002
Total fat (E%)	25-40	41.2	7.0	44	40.6	7.1	45.6	43.2	6.6	38.9	0.153
SFA (E%)	≤10	16.9	4.0	2.7	16.8	4.3	3.5	17.2	3.1	0	0.682
MUFA (E%)	10-20	15.0	3.0	93.3	14.6	2.8	93.0	16.5	3.1	94.4	0.031
PUFA (E%)	5-10	5.4	1.8	57.3	5.3	1.9	52.6	5.9	1.5	72.2	0.196
Total carbohydrates (E%)	45-60	40.4	8.0	26.7	41.9	7.9	33.3	35.9	6.8	5.6	0.004
Sucrose (E%)	≤10	12.2	5.7	36.0	13.7	5.5	24.6	7.6	3.7	72.2	<0.001
Dietary fibre (g/d)	25-35	20.2	6.4	16.0	20.5	6.6	19.3	19.2	5.6	5.6	0.435
Dietary fibre (g/MJ)	≥3	2.1	0.7	13.3	2.2	0.7	17.5	1.7	0.4	0	<0.001
Protein (E%)	10-20	15.8	3.1	92.0	15.3	2.9	93.0	17.3	3.2	83.3	0.027
Alcohol (E%)	≤5	2.6	4.3	77.3	2.3	3.8	80.7	3.7	5.5	66.7	0.347

p-value denotes the difference between women and men. A p-value <0.05 is set as statistically significant. NNR: Nordic Nutrition Recommendation values, RI: recommended intake according to the NNR.

Analysis of total fat intake showed that 54.4% of the women and 61.1% of the men had a consumption that was over the upper RI limit (i.e. 40 E%), and no subjects being under the lower RI limit (i.e. 25 E%). Two of all the subjects had a SFA intake within the RI of ≤ 10 E%. None of the men complied with the RI for SFA. The MUFA intake complied very well with the NNR. The RI of PUFA was reached by 52.6% of the women and by 72.2% of the men. No significant differences between the genders were observed for the intake of fat.

The adherence to the RI for the intake of total carbohydrates (expressed as E%) was found to be low. Women showed a higher mean intake of sucrose (13.7 ± 5.5 E%) than the RI whereas men showed a mean intake (7.6 ± 3.7 E%) within the recommendations. Both total carbohydrate and sucrose intake were significantly higher in women compared to men. The RI of 25–35 g of dietary fibre per day was met by 19.3% of women and by 5.6% of men. When fibre intake was adjusted for energy intake (expressed as g/MJ), 17.5% of women and none of men met the recommended intake of 3 g/MJ, with a statistically significant difference in mean fibre intake between the genders (*p* < 0.001).

The compliance to the RI for protein was relatively high and a significantly higher mean protein intake among men was observed compared to women (p = 0.027).

### Micronutrients

Analysis of micronutrient intake data (Table 3) showed that vitamin D intake was low: 29.8% of the women and 27.8% of the men reached the AR of 7.5 µg. The adherence to the AR for vitamin C intake was rather low whereas the adherence to all other calculated vitamin and mineral intakes was high with results ranging from 70 to 100%. The extent of adherence was very similar between men and women for all micronutrients except for iron. All men had a mean iron intake above the AR whereas 70.2% of the women reached the AR. Mean sodium intake was higher than the RI for all men and 24.6% of the women had a sodium intake within the RI of less than 2.4 g/day.

**Table 3.** Mean daily micronutrient intake in relation to NNR values.

Micronutrients	Women (n = 57)				Men (n = 18)			
	NNR (AR)	mean	SD	% subjects reaching AR	NNR (AR)	mean	SD	% subjects reaching AR
Retinol Equivalent (RE)	500	874.0	371.1	80.7	600	856.4	324.9	72.2
Vitamin D (µg)	7.5	6.5	3.2	29.8	7.5	6.2	3.5	27.8
Tocoferol (mg)	5	13.0	3.9	98.3	6	15.2	4.2	100
Thiamin B1 (mg)	0.9	1.4	0.5	86.0	1.2	1.6	0.5	100
Riboflavin (mg)	1.1	1.4	0.4	80.7	1.4	1.8	0.5	83.3
Niacin Equivalent (NE)	12	35.3	9.4	100	15	45.3	8.9	100
Vitamin B6 (mg)	1.1	1.9	0.6	73.7	1.3	2.0	0.5	100
Vitamin B12 (µg)	1.4	4.5	2.4	98.3	1.4	6.8	2.6	100
Folate (µg)	200	253.7	62.4	77.2	200	299.6	81.1	88.9
Vitamin C (mg)	50	83.8	54.4	70.2	60	64.9	25.9	55.6
Calcium (mg)	500	828.9	280.6	87.7	500	1124.1	377.2	94.4
Phosphor (mg)	450	1353	301	100	450	1778	360	100
Iron (mg)	6^a^, 10	9.1	2.6	70.2	7	11.2	3.0	100
Sodium (g)	<2.4^b^	3.1	1.0	24.6^b^	<2.4^b^	4.3	0.8	0^b^
Zinc (mg)	5	10.4	3.0	96.5	6	14.9	3.1	100
Selenium (µg)	30	45.4	19.8	79.0	35	57.7	18.1	88.9

NNR: Nordic Nutrition Recommendation values, AR average requirement.

^a^ for postmenopausal women.

^b^ RI is used for sodium intake since AR is not available.

Table 4 shows the nutrient density (per 10 MJ) for micronutrients of our data, NNR and the average diet in Sweden (Amcoff et al., 2012). The nutrient density of vitamin D, folate and iron was clearly too low when compared to the reference values of NNR. However, mean nutrient density of these three micronutrients was also low in the average diet in Sweden. The nutrient density of vitamin C and B6 was significantly lower in men than women whereas nutrient density of sodium and zinc was significantly higher in men compared to women (Table 4). For all other micronutrients, the nutrient density was similar between men and women.

**Table 4.** Nutrient density (per 10MJ) for micronutrients.

Micronutrients	NNR^a^	Average diet in Sweden^b^	Women (n = 57)		Men (n = 18)		p-value
			mean	SD	mean	SD	
Retinol Equivalent (RE)	800	1117	954.5	380.7	779.9	304.6	0.054
Vitamin D (µg)	14.0	8.8	7.2	3.7	5.7	3.6	0.142
Tocoferol (mg)	9	15.8	14.2	3.8	13.7	3.7	0.608
Thiamin B1 (mg)	1.2	1.5	1.5	0.6	1.4	0.4	0.378
Riboflavin (mg)	1.4	1.9	1.5	0.4	1.7	0.5	0.221
Niacin Equivalent (NE)	16	43	38.8	10.1	40.9	7.0	0.349
Vitamin B6 (mg)	1.3	2.5	2.0	0.6	1.8	0.3	0.026
Vitamin B12 (µg)	2	6.9	5.0	2.8	6.3	2.7	0.105
Folate (µg)	450	349	280.5	67.2	268.2	70.0	0.516
Vitamin C (mg)	80	132	93.8	60.3	58.2	20.8	<0.001
Calcium (mg)	1000	1114	895.6	257.6	1013.2	360.8	0.213
Phosphor (mg)	800	1697	1479.2	237.0	1601.5	312.3	0.140
Iron (mg)	16	13.1	9.9	2.5	10.0	2.2	0.909
Sodium (g)	na	na	3.3	0.8	3.9	0.9	0.014
Zinc (mg)	12	13.1	11.4	2.9	13.4	2.7	0.010
Selenium (µg)	57	58	50.9	23.6	51.8	15.6	0.851

p denotes difference between women and men. A p-value <0.05 is set as statistically significant. na: not available.

^a^ reference values for nutrient density by Nordic Nutrition Recommendation.

^b^ based upon a national dietary survey published by Swedish National Food Agency.

## Discussion

In this study we evaluated the nutrient intake of 75 subjects with abdominal obesity living in southern Sweden and studied their adherence to the reference values of NNR. Results showed the adherence to NNR was rather low with a high total fat intake, particularly high intake of saturated fatty acids, high salt consumption, and very low dietary fibre and vitamin D intake.

Lifestyle diseases, including obesity, primarily results from energy imbalance or overconsumption. Our results showed that the mean daily energy intake was higher compared to the Swedish adult population (Amcoff et al., 2012). However, the energy distribution between the different macronutrients is of even more importance than the total energy intake.

It was found that the RI range for total fat was exceeded by 56% of the subjects. The consumption of total fat in the present study is about 7% higher than for the average Swedish population (i.e. 34.2 ± 6.4 E%) (Amcoff et al., 2012). However, the fat quality is of significance when analysing the total fat intake.

Our results show a clear overconsumption of SFA. This is in line with previous data showing that there is a marked high intake of SFA in subjects having obesity or MetS (Jonsdottir et al., 2013). The intake of MUFA matched the RI well, whereas the RI of PUFA was met by approximately 60%.

High intake levels of SFA have been associated with up to 13% increase in mortality, whereas replacement of SFA with PUFA or MUFA reduce the mortality with 19% and 11%, respectively (Clifton and Keogh, 2017; Hooper et al., 2015). Reduction of the PUFA intake concomitant with an elevated consumption of SFA has been indicated to increase the risk for CVD in several studies (Grundy, 2016; Sacks et al., 2017). Data have also suggested that a shift towards PUFA while reducing SFA consumption decreases the incidence of CVD (Mozaffarian et al., 2010). Other studies have shown that increasing the PUFA intake reduces liver fat and improves metabolic status and reduces inflammation independent of body weight (Bjermo et al., 2012).

The carbohydrate consumption among the subjects of this study was on average similar to the Swedish national average (i.e. 43.6 ± 7.3 E%) (Amcoff et al., 2012). The relative low intake of the carbohydrate might demonstrate a shift towards fat consumption which is in line with previous Nordic diet studies (Kanerva et al., 2014). Studies have indicated that a higher total carbohydrate intake correlates inversely with obesity (Kaartinen et al., 2016; Kanerva et al., 2013). It may therefore be beneficial for subjects with obesity to replace a fraction of the total fat consumption by complex carbohydrates.

Our results show a low adherence to the RI of dietary fibre of 25–35 g/day or at least 3 g/MJ and this is in agreement with a previous study conducted in the Nordic countries (Jonsdottir et al., 2013). Diets enriched for fibre content including whole grain have been associated with health-promoting effects on clinical outcomes as insulin resistance, mortality from CVD and incidence of the MetS (McKeown et al., 2004; Magnusdottir et al., 2014; Sahyoun et al., 2006). A dietary intervention study with Nordic foods according to the NNR showed that increased whole grain intake, measured by plasma alkylresorcinols, was associated with reduced blood lipid levels (Magnusdottir et al., 2014). The consumption of high fibre diet by subjects with MetS has also demonstrated to reduce blood pressure, improve blood lipid profile, and reduce inflammatory burden (Brader et al., 2013; Uusitupa et al., 2013). Moreover, increased dietary fibre intake has been correlated to reduced overall mortality in a Danish cohort (Olsen et al., 2011). Since our results showed a low daily fibre intake, subjects with obesity should be advised increasing the intake of fibre-rich foods such as of whole grains, fruits, berries, vegetables, pulses, nuts and seeds.

More than 70% of our subjects reached the AR for all micronutrients except for vitamin D. Mean nutrient density for vitamin D was also lower than reference value by NNR and in the average diet in Sweden. Previous data from a Nordic population with the MetS showed that 45% had levels under the RI of 10 µg and 8% were under the lowest level of intake of 2.5 µg (Brader et al., 2014). Several studies have demonstrated a relation between reduced plasma vitamin D levels and higher prevalence of obesity independent of latitude and age (Pereira-Santos et al., 2015). Larger doses of vitamin D supplementation may be required to adjust the levels in subjects with obesity compared to lean individuals, in part due to possible effects of sequestration in adipose tissue (Pourshahidi, 2015). Vitamin D deficiency is very common among the population living in the Nordic countries due to low sun exposure (Lamberg-Allardt et al., 2013).

Elevated intake of sodium has been robustly linked to elevated blood pressure and CVD, including increased mortality (He and MacGregor, 2011). Reduction of sodium intake has been demonstrated to have beneficial effects on hypertension and therefore cardiovascular events (Aburto et al., 2013). Almost all subjects included in our study had higher sodium intake levels than recommended by NNR. Results from the Swedish national governmental survey showed a mean sodium intake for men and women of 3.6 and 2.7 g/d, respectively, which is higher than the recommendations (Amcoff et al., 2012). This is also in agreement with our results where nutrient density of sodium in men is significantly higher than in women. Since obesity on its own is a risk factor for CVD, it is of high importance to decrease the sodium intake in subjects with obesity. Suggestions to lower sodium intake include e.g., to avoid consumption of processed foods, to avoid extra salting during cooking and to replace salt by herbs.

In general, our results show that men consume significantly less dietary fibre and vitamin C than women. These results might suggest that men eat lower daily amounts of fruits and vegetables compared to women. On the other hand, sugar intake of men in our cohort was significantly lower compared to women and can be considered as a health advantage. From these results, it is difficult to confirm that women have a healthier eating pattern, as stated by the results from the Swedish governmental dietary survey (Amcoff et al., 2012). Further studies are needed to assess dietary quality among adults with obesity by evaluating specific food groups and using a dietary quality score in the Nordic countries.

Food diaries are often used as an indicator of the usual intake of nutrients. The usual intake of nutrients is an important factor when evaluating food intake and is defined as the average intake over of longer period of time. Our results might have been more correct when using a food diary for a longer period and/or when applying another method of dietary assessment. Also, a frequency questionnaire assessing the intake of specific food groups should have been of interest to evaluate food habits and patterns in this study.

The number of recruited participants is based upon a power calculation using body weight and SAD as primary endpoint in the long-term weight management intervention study. The small number of participating subjects, living in the Southern part of, is a limitation of the study and our results do not reflect the Swedish population with obesity. An increased number of subjects, especially males, might have increased the reliability of the results.

Several dietary reference values are available within NNR (Nordic Nutrition Recommendations, 2012). They are used to ensure optimal nutrition and body function and contribute to a reduced risk of diet-related lifestyle diseases. Recommended intakes are mainly used as guidelines for dietary planning for various groups whereas requirements are strongly suggested to use to evaluate the probability of inadequacy of nutrients intake (Nordic Nutrition Recommendations, 2012). In our study, we have used AR and nutrient density for all available micronutrients and RI for the macronutrients and sodium. NNR are intended for the general healthy population and not for specific groups such as subjects with obesity. Furthermore, it is important to mention that the reference values are valid for assessment at group level and do not show that requirements are met for each individual.

In summary, the nutrient intake in our Swedish subjects with abdominal obesity compared to the recommendations from NNR was rather low with a high total fat intake, particularly too high intake of SFA, and very low dietary fibre intake. Almost all subjects had a sodium intake above the recommendation and a mean low vitamin D intake. Further efforts from policy making institutions in the Nordic countries are clearly needed in order to promote better nutrient intake and nutritional quality for this vulnerable group. Improved dietary quality can be reached by a promoting e.g., the consumption of vegetables, fruits and berries, fish, and nuts and by limiting e.g., the consumption of processed foods and red meat, and foods and drinks with added sugars.

## Supplemental Material

sj-doc-1-nah-10.1177_02601060221105751 - Supplemental material for Nutrient intake and adherence to the Nordic nutrition recommendations in a Swedish cohort with abdominal obesity Supplemental material, sj-doc-1-nah-10.1177_02601060221105751 for Nutrient intake and adherence to the Nordic nutrition recommendations in a Swedish cohort with abdominal obesity by Åsa Sedin, Mona Landin-Olsson and Lieselotte Cloetens in Nutrition and Health
